# *“Trying to write a story together”*: general practitioners’ perspectives on culturally sensitive care

**DOI:** 10.1186/s12939-024-02200-9

**Published:** 2024-06-06

**Authors:** Robin Vandecasteele, Lenzo Robijn, Peter A. J. Stevens, Sara Willems, Stéphanie De Maesschalck

**Affiliations:** 1 Faculty of Medicine and Health Sciences, Department of Public Health and Primary Care, Research Group Equity in Health Care, Ghent University, University Hospital Campus, C. Heymanslaan 10, Ghent, 9000 Belgium; 2 Department of Sociology, Faculty of Political and Social Sciences, Ghent University, Sint-Pietersnieuwstraat 41, Ghent, 9000 Belgium; 3 Faculty of Medicine and Health Sciences, Department of Public Health and Primary Care, Quality & Safety Ghent, Ghent University, University Hospital Campus, C. Heymanslaan 10, Ghent, 9000 Belgium; 4 Centre for the Social Study of Migration and Refugees, Ghent University, H. Dunantlaan 2, Ghent, 9000 Belgium

**Keywords:** Culturally sensitive care, Cultural preferences, Perceived responsibilities, Adaptation of Care, Cultural Empathy, Cultural Curiosity

## Abstract

**Background:**

Culturally sensitive care is integral to effective and equitable healthcare delivery, necessitating an understanding and acknowledgment of patients’ cultural needs, preferences, and expectations. This study investigates the perceptions of cultural sensitivity among general practitioners (GPs), focusing on their intentions, willingness and perceived responsibilities in providing care tailored to cultural needs.

**Methods:**

In-depth interviews were conducted with 21 Flemish GPs to explore their perspectives on culturally sensitive care. Data analysis followed a conventional qualitative content analysis approach within a constructivist framework. A coding scheme was developed to identify recurring themes and patterns in the GPs’ responses.

**Results:**

Findings reveal that culturally sensitive care provision is perceived as a multifaceted process, initiated by an exploration phase where GPs inquire about patients’ cultural needs and preferences. Two pivotal factors shaping culturally sensitive care emerged: patients’ specific cultural expectations and GPs’ perceived responsibilities. These factors guided the process of culturally sensitive care towards three distinct outcomes, ranging from complete adaptation to patients’ cultural requirements driven by a high sense of responsibility, through negotiation and compromise, to a paternalistic approach where GPs expect patients to conform to GPs’ values and expectations. Three typologies of GPs in providing culturally sensitive care were identified: genuinely culturally sensitive, surface-level culturally sensitive, and those perceiving diversity as a threat. Stereotyping and othering persist in healthcare, underscoring the importance of critical consciousness and cultural reflexivity in providing patient-centered and equitable care.

**Conclusions:**

This study emphasizes the significance of empathy and underscores the necessity for GPs to embrace the exploration and acknowledgement of patients’ preferences and cultural needs as integral aspects of their professional role. It highlights the importance of shared decision-making, critical consciousness, cultural desire and empathy. Understanding these nuances is essential for enhancing culturally sensitive care and mitigating healthcare disparities.

## Background

In contemporary societies, multiculturalism and superdiversity have become the norm [1]. The provision of culturally appropriate and sensitive care has therefore evolved towards a fundamental aspect of health care delivery. This evolution is underscored by research demonstrating its pivotal role in equitable care and recognizing it as an essential component of the CanMeds framework, one of the most prominent frameworks used in medical education and practice delineating the required roles and competencies of physicians in today’s complex healthcare environment [2–4]. Health care providers are required to explore and, where applicable, adapt to specific needs and expectations of patients with diverse ethnic backgrounds in order to overcome cultural and linguistic barriers that may arise during intercultural consultations [5, 6].

In intercultural care encounters, it is necessary to recognize that minority patients – individuals whose ethnic, cultural, religious or linguistic background differs from the dominant population in a certain area – frequently exhibit variations in health beliefs, values, preferences and behaviors [7, 8]. Disregarding culturally specific needs has been identified as a key factor contributing to ethnic healthcare disparities [9, 10]. Previous research demonstrated that the failure to tailor healthcare practices to the needs of patients from diverse ethnic backgrounds can lead to various adverse consequences. These implications encompass diminished levels of patient satisfaction, increased mistrust in providers, lower therapy adherence, feelings of disempowerment and, ultimately, overall suboptimal health outcomes [11–13]. The imperative to address culturally specific patient needs is further underscored by the existing disparities in health outcomes observed among certain ethnic minority groups, who disproportionately experience higher risk for infectious diseases [14], higher premature death rates from heart disease [15] or who display increased prevalence of anxiety and depression [16]. Recognizing the central role of healthcare providers, particularly general practitioners (GPs), in shaping the health care experiences and outcomes of ethnic minority patients, it is widely acknowledged that substantial efforts are necessary to enhance providers’ intercultural awareness and skills [17, 18]. GPs, as primary points of contact for individuals seeking healthcare, assume a pivotal role in ensuring effective and equitable medical care.

Consequently, the notion of cultural sensitivity or competence was introduced as a strategy to tackle the above mentioned ethnic health disparities [3]. Culturally sensitive care refers to the ability of healthcare professionals to provide equitable and high-quality care to all patients, regardless of ethnicity, culture or language proficiency [9]. By recognizing, acknowledging and integrating the significance of culture in patients’ health beliefs and behavior, healthcare provision can be tailored to cater to culturally specific requirements [3, 19, 20].

The terms culturally sensitive care and culturally competent care are often used interchangeably in the literature and essentially encompass the same core concept [21, 22]. Nonetheless, a nuanced distinction is evident between cultural sensitivity and competence, wherein cultural sensitivity entails a heightened awareness of one’s own culture and recognition of, as well as respect for, the cultural background of others [23–25]. Therefore, culturally sensitive care necessitates an understanding of both cultural similarities and differences in how people perceive health and illness, communication with healthcare providers and, consequently, tailoring care approaches accordingly. Conversely, the concept of cultural competence has attracted criticism for its tendency to portray culture as static, overemphasize cultural differences and fails to consider the impact of healthcare providers’ personal cultural values and professional cultural norms [26, 27]. Moreover, it often overlooks the inherent diversity within cultural groups and neglects the intersection of other dimensions of patient identity, frequently resulting in stereotypical assumptions [27, 28]. Additionally, related terms such as ‘cultural humility’, ‘cultural awareness’ and ‘cultural safety’ are frequently employed to describe this notion, each highlighting distinct aspects [3, 27, 29–31].

In practice, a culturally sensitive approach entails GPs identifying patients’ individual needs by, for instance, exploring patients’ ideas, concerns, expectations and reasons for seeking consultation or assessing patients’ language abilities [6, 32]. It also requires clinicians to be able to critically reflect on their own beliefs and values (i.e. cultural reflexivity [33]). Furthermore, cultural sensitivity involves a willingness to understand the perspectives and traditions of culturally diverse patients, considering the intersecting factors of their identities, such as education, socio-economic status, and gender identity. This allows providers, where necessary and possible, to adapt diagnostic and treatment policies to patients’ unique cultural contexts [6, 34]. This approach enables GPs to provide more patient-centered, tailored and effective care responsive to the diverse needs and experiences of all patients.

GPs’ cultural sensitivity and competence have been associated with a multitude of positive outcomes [23]. These include heightened levels of trust in providers [35], increased patient satisfaction and perceived quality of care [36, 37], patients engaging more in information-sharing and information-seeking behaviors [37], more effective communication [20] and improved therapy adherence [38–40]. The cumulative effects of these advantages culminate in overall improved health outcomes [23].

Because of these benefits, there has been a growing emphasis on integrating cultural sensitivity into the medical education of future practitioners, as well as their continuous training throughout their professional development [41]. Yet, the majority of medical schools either inadequately or superficially addresses this topic, resulting in inconsistent levels of instruction [41, 42]. Consequently, the overall state of cultural sensitivity in medical education is often deemed insufficient and fragmented [43]. Moreover, numerous intervention strategies designed to enhance GPs’ cultural sensitivity have been developed and tested [44, 45]. However, research results remain inconsistent on the impact of training on physicians’ behavior in intercultural consultations [18, 24, 44, 46–48]. Whereas some interventions resulted in improved practitioner cultural knowledge and attitudes, minimal evidence exists supporting positive intervention effects regarding GPs’ behavioral changes, minority patients’ satisfaction or health outcomes.

Furthermore, very few indications of culturally sensitive care have been observed in general practice research [6, 49–51]. The lack of implementing culturally sensitive strategies may be due to the inherently complex work environment typical for primary care, as interactions with patients and patients’ relatives can be particularly challenging, often without clearly defined problems or standard solutions [52]. Even with specific cultural knowledge, considerable nuance is advised when applying this knowledge in specific situations and interactions with patients from diverse backgrounds. Therefore, continuous critical reflection and reassessment of personal perceptions, biases, competencies and how they affect patient interactions is required [10, 52].

Another possible explanation for the lack of culturally sensitive strategies in practice is that healthcare students and providers consider cultural sensitivity a “soft science” or inferior to basic science or clinical knowledge [9, 53, 54]. This perspective persists even though cultural sensitivity is inherently tied to professional competence and is integral to GPs’ CanMed roles [4, 55]. Shepherd and colleagues [56] also found that practicing health care professionals scarcely acknowledge the need for cultural awareness and critical reflexivity of one’s own culture, despite being central components of cultural sensitivity [21]. Additionally, Dauvrin and Lorant [5] reported that GPs do not feel responsible for adapting care in accordance with patients’ cultural preferences. This responsibility was predominantly attributed to patients themselves. GPs’ apathy and resistance towards the subject, along with high reported levels of unpreparedness and unawareness [50], may be important contributors to the lack of cultural sensitivity in today’s general practice. However, further research is needed to determine GPs’ moral reasoning, perceptions of culturally sensitive care and its absence in today’s primary care [23, 50, 56].

Moreover, understanding of these issues within the European context is limited due to inadequate data availability [57, 58]. The Flemish context, representing the largest segment of Belgium, is of particular interest due to its extensive history of immigration. Studies have also revealed that certain discriminatory and inequitable care practices are more prevalent in Belgium compared to other European nations [16]. Ascertaining the views and experiences of GPs regarding culturally sensitive care provision is of profound importance in advancing our understanding of cultural sensitivity in health care, and the lack thereof [23, 56]. Such insights from GPs have been notably limited in the existing literature and, therefore, this study aims to qualitatively explore GPs’ perceptions regarding culturally sensitive care, their intentions to implement culturally sensitive strategies, their willingness to adapt care provision based on cultural considerations, and their perceived professional responsibilities.

## Methods

### Design

This study adopted a qualitative approach under a constructivist perspective to gain insight into the perspectives and experiences of Flemish GPs regarding culturally sensitive care. Recognizing the multifaceted nature of cultural sensitivity in healthcare provision, this methodological approach allows for a comprehensive exploration of the complex interplay between cultural factors and healthcare practices. By emphasizing the subjective realities and contextual interpretations of GPs, the study aims to uncover how cultural considerations shape their perceptions and behaviors in clinical settings.

Within this approach, our study utilized in-depth, semi-structured interviews. A topic guide was developed to comprehensively explore all pertinent aspects concerning GPs’ attitudes, intentions, and perceived responsibilities in delivering culturally sensitive care. Interview questions were based on the existing literature within the scope of our research objectives, recommendations and identified knowledge gaps. Developing the topic guide was a collaborative process involving all authors, with iterative revisions to refine its content and scope. Additionally, two pilot interviews were conducted with GPs experienced both in cross-cultural care and qualitative research to assess the relevance of the identified topics and evaluate the interview questions’ quality and clarity.

### Participants

A purposive sampling strategy was employed to select participants for this study, drawing from GPs who participated in our earlier study [51]. Our prior study investigated GPs’ consulting behavior and effectiveness in intercultural care encounters, utilizing online video recorded consultations with ethnic minority patients and comparing with their behavior in consultations with ethnic majority patients. GPs who had participated in the prior study and expressed willingness to engage in follow-up interviews, were contacted through email and invited to provide additional insights on culturally sensitive care. GPs were included for selection based on the criteria of gender, years of experience and practice characteristics, aiming for a diverse and more representative sample, including GPs’ exhibiting both exemplary and substandard performances in interactions with simulated patients from an ethnic minority background. Since the prior study’s participant pool primarily comprised Flemish majority GPs, our capacity to attain ethnic diversity in the current sample was severely constrained and consequently not pursued. The interview session scheduling was coordinated according to the respondents’ preferences.

In total, 21 GPs participated in this study, comprising eight female and 13 male GPs (Table 1). The age range of participants was 27–64, with a mean age of 45.1 years. Most GPs in our sample reported frequent consultations with ethnic minority patients, ranging from a weekly to a daily basis. Further, most respondents had not undergone any culturally sensitive training intervention or program. Further demographic details are presented in Table 1.

**Table 1:** GP characteristics

General Practitioners	*N*/M
Gender	
female	8
male	13
Age	45.1
min	27
max	64
Years of experience	17.19
min	1
max	37
Practice composition	
solo practice	4
solo + GP trainee	5
2 GPs	3
2 GPs + GP trainee	1
group practice	8
Frequency consulting ethnic minority patients	
(almost) never	1
once or a few times a month	1
weekly	6
few times a week	6
daily	7
Followed culturally sensitive training	
yes	2
no	19

### Procedure

This study was reviewed and approved by the Ethics Committee of University Hospital Ghent (EC registration number: BC-08924), and all participating GPs signed an informed consent before conducting the interviews. Respondents were assured that participation was voluntary and anonymous, that data was stored securely, and that they could stop the interview at any time if they chose to do so.

In addition, during the introductory briefing covering the study’s objectives, ethical considerations, and interview procedures, participants were provided with a concise description of cultural sensitivity to ensure a common understanding. Data were collected between April and September 2022, with interviews lasting between 30 and 60 min. Audio recordings of the interviews were transcribed verbatim. Data collection continued until theoretical saturation, the point at which no new information or themes emerged from the interviews, was achieved.

### Data analysis

Data analysis was conducted using Nvivo, version R1. The analysis followed a conventional qualitative content analysis approach, as described by Hsieh and Shannon [59]. As current research and evidence of GPs’ perceptions regarding culturally sensitive care is limited, the adoption of this approach is considered suitable. Qualitative content analysis allows for the systematic exploration of large quantities of data, facilitating in-depth examinations of participants’ experiences and perspectives and has, therefore, become a standard methodology in health research, effectively aligning with our research objectives [59, 60]. This methodology aims to develop a coding scheme by systematically organizing data into meaningful categories, which can be employed in further research in similar settings or fields, thereby enhancing the reliability and comparability of findings within similar contexts or fields.

Two independent coders (RV and LR) engaged in the coding of three randomly selected interviews, subsequently comparing their respective coding to establish an initial coding framework. The coding framework underwent a comprehensive discussion and validation process involving all authors before further application. Once an agreement was achieved, the established coding framework was employed to code all interviews systematically. This coding procedure was consistently upheld as the same pair of coders independently coded the entirety of the dataset. The coders compared their findings and engaged in further discussions to ascertain agreement on the data coding and the final iteration of the coding framework. This rigorous iterative process culminated in a mean kappa coefficient of 0.93, underscoring the ‘almost perfect’ intercoder reliability of the analysis [61] and resulted in the creation of a coding scheme revealing five distinct categories: the need for cultural sensitivity, patients’ cultural preferences, culturally sensitive strategies and activities, perceived responsibilities and perceived ability to adapt care provision (Table 2).

**Table 2:** Coding scheme

Category	Description	Subcategory description	Applicability	Example
*Need for cultural sensitivity*	Codes demonstrating GPs’ recognition of gaps, challenges, or areas for improvement in the delivery of equitable care, as well as the acknowledgment of the importance and benefits of integrating cultural sensitivity into clinical practice. This category aims to capture GPs’ reflections on the necessity of adopting culturally sensitive approaches to address the diverse cultural needs, preferences, and concerns of their patient populations effectively.	Subcategories are stratified according to the degree to which participants deem culturally sensitive care provision as necessary.	Code instances where GPs express perceptions, experiences, or insights related to the need for cultural sensitivity in healthcare delivery. Consider the multifaceted nature of cultural sensitivity, including its implications for communication, patient care, health outcomes, and healthcare disparities. Note variations in the perceived need for cultural sensitivity across different healthcare settings, patient populations, and geographic regions, as well as the implications for policy and practice interventions aimed at promoting culturally competent care provision.	Social determinants of health Patient-centeredness Equity vs. Equality
*Patients’ cultural preferences*	This category includes the cultural values, norms, beliefs, practices and preferences that GPs observe and experience among their patient populations. It involves identifying and understanding the specific cultural beliefs, practices and preferences expressed by patients during healthcare encounters, as perceived by GPs. This category aims to capture the insights GPs gain regarding their patients’ cultural backgrounds and preferences, and how these insights fit in and influence the provision of culturally sensitive care.	Subcategories are divided as follows: cultural perceptions of illness, dietary preferences, gender-related norms and preferences, communication strategies, medication preferences and stereotypes.	Code instances where GPs express observations or insights about the cultural preferences and needs of their patient populations. Consider the implications of these cultural preferences on the provision of culturally sensitive care, including the need for tailored communication strategies, collaborative decision-making approaches, and culturally competent care planning. Note variations in cultural preferences across different patient populations and healthcare contexts, and how GPs adapt their practices to accommodate these cultural differences effectively.	Gender concordance Role of family in consultations Requesting antibiotics
*Culturally sensitive strategies and activities*	Codes in this category describe the diverse range of behaviors implemented by GPs to deliver culturally sensitive care. It involves identifying and understanding the specific actions, techniques, and initiatives undertaken by GPs to recognize, respect, and respond to the cultural diversity of their patients. This category aims to capture the breadth and depth of culturally sensitive approaches utilized by GPs in their clinical practice to promote health equity, improve patient-provider communication, and enhance healthcare outcomes among culturally diverse populations.		Code instances encapsulating the diverse array of strategies and activities articulated by GPs, ranging from broad conceptual frameworks to specific practical techniques.	Exploring patients’ needs Inquiring about gender preferences for referral Cultural curiosity Critical self-reflection
*Perceived responsibilities*	This category encompasses perceptions and expectations regarding the roles, duties, and obligations of GPs in delivering culturally sensitive care, as well as perceptions about the roles patients should play in this process. It involves stakeholders’ beliefs about the specific functions, tasks, and responsibilities that GPs should fulfill in addressing the cultural needs, concerns, and preferences of their diverse patient population, and the corresponding roles patients are perceived to play in achieving culturally sensitive healthcare. This category aims to capture the perceived scope, boundaries, and accountability associated with GPs’ responsibilities in culturally competent healthcare delivery.	Subcategories are divided by distinct responsibilities attributed to GP and/or patient.	Note variations in expectations across patients’ specific needs, case examples, cultural contexts, and patient populations to capture the range of perceived responsibilities associated with culturally sensitive care provision.	Tailor therapy recommendations to cultural needs Expect patient assimilation
*Perceived ability to adapt*	This category encompasses perceptions regarding the extent to which general practitioners demonstrate flexibility and capability in adjusting their care practices to meet the cultural requirements, preferences, and expectations of patients from diverse cultural backgrounds. It involves the perceived readiness, willingness, and efficacy of GPs in adapting their communication styles, treatment plans, and overall approach to better align with the cultural context of their patients. This category seeks to capture the subjective assessments and beliefs of stakeholders regarding the adaptability of GPs in providing culturally sensitive care.	Subcategories are divided by the extent of the expressed ability to adapt, varying from very low to very high perceived ability.	Code instances where stakeholders express opinions, observations, or experiences related to GPs’ perceived adaptability or inflexibility in addressing cultural considerations in their care provision. Consider the context, nuances, and specific dimensions of adaptability (e.g., communication, treatment, cultural awareness) when assigning relevant codes. Note variations in perceptions across different cultural groups, patient-provider dynamics, and healthcare settings to capture diverse perspectives on GPs’ ability to adapt in culturally sensitive care provision. Lastly, by splitting the categories of ability and responsibility, a clear distinction can be made regarding whether GPs feel morally responsible to tailor care to patients’ needs and how capable they ultimately are in adapting care provision.	Not knowing cultural expectations Views on medical guidelines/bioclinical models

## Results

**Fig. 1:**
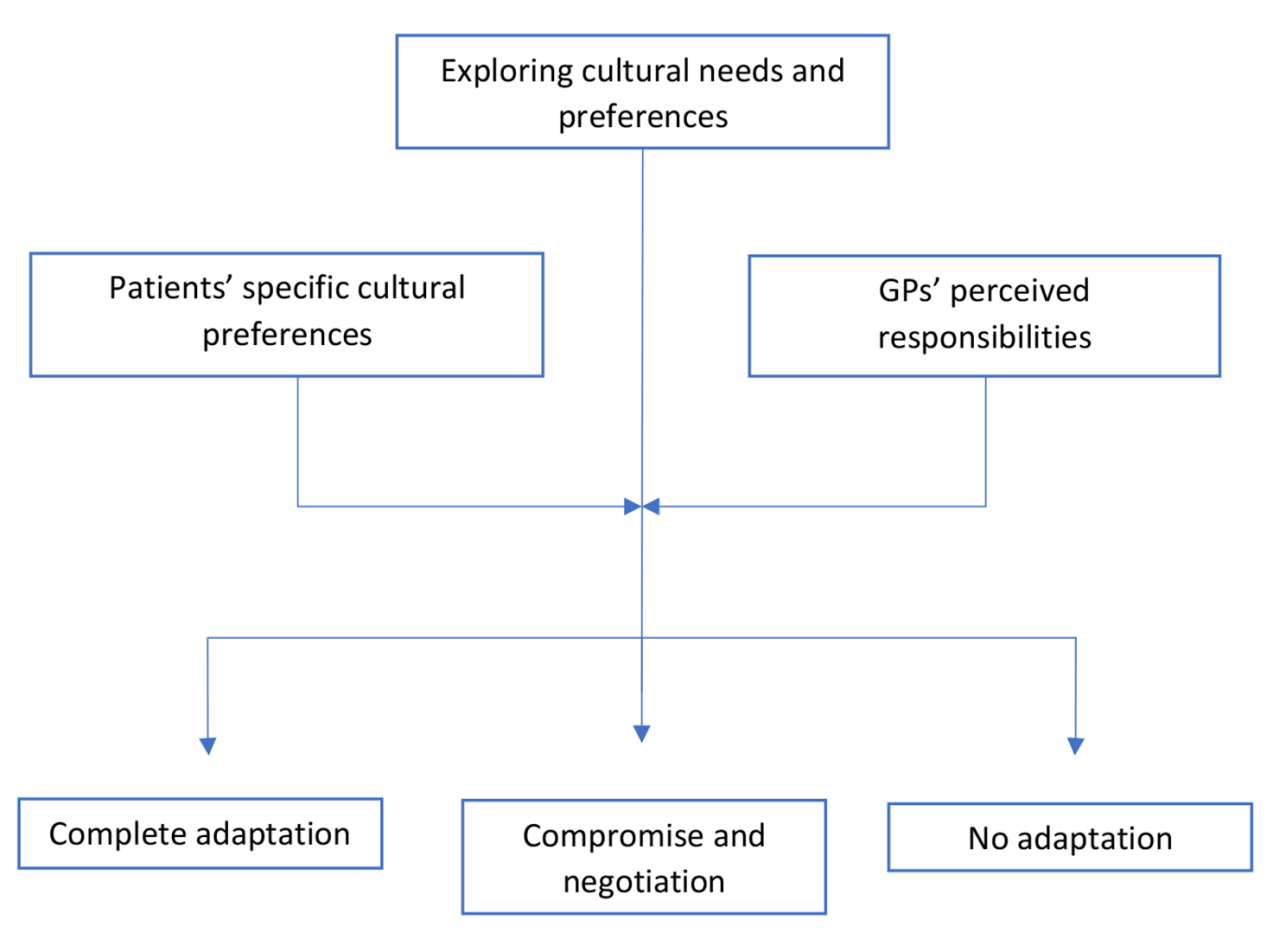
The process of culturally sensitive care

The following section delves into GPs’ conceptualization of culturally sensitive care, derived from the interpretation of the coding scheme utilized in our analysis. First, we describe GPs’ perspective on cultural sensitivity, characterizing it as an intricate and dynamic *process*. Second, we describe how this process is shaped by two pivotal factors: (a) patients’ specific cultural preferences and (b) the responsibilities GPs attribute to themselves (Fig. 1).

### Culturally sensitive care as a process

The majority of GPs in our sample did recognize the need for culturally sensitive care. According to our participants, customizing to individual patient needs, including cultural preferences and expectations, is necessary in order to attain an equitable care provision. Acknowledging the diverse contexts and backgrounds of patients, care should ideally be tailored to their respective realities and possibilities. This approach involves implementing differential treatment characterized by an equal measure of respect for and interest in each patient, with the ultimate goal of achieving equitable outcomes.

Our data revealed several health-related cultural variations between GPs in our sample, consisting exclusively of ethnic majority practitioners, and their diverse patient populations. Notably, discussions on cultural sensitivity mostly revolved around patient interactions with individuals from a Moroccan-Maghreb or Turkish background, two prominent ethnic, (predominantly Muslim) minority groups within Flanders, Belgium. GPs primarily addressed commonly occurring, somewhat stereotypical examples, such as culturally specific eating habits, preferences regarding healthcare providers’ gender, religious traditions (e.g. Ramadan, a month-long Islamic fasting period characterized by daylight abstinence from food and drink), and the expression of symptoms. However, some respondents were observed to value genuine cultural curiosity, expressing a deeper interest in understanding cultural nuances beyond these surface-level differences.

GPs note that a culturally sensitive approach requires a substantial additional investment of time and effort, often scarcely available in general practice. This scarcity contributes to the perception of GPs in our study that culturally sensitive care is a process of ongoing pursuit, with GPs striving to achieve care provision as culturally sensitive as restrictions allow.

*“I think that we should strive to achieve the same quality of care, especially in terms of results, for everyone. Now, I do believe that it is a pursuit and that it may not necessarily be realized. But I think the differences mainly lie in the process because… yes, there are multiple ways to reach Rome, and the same path is not equally good for everyone.”* (male GP, 29).

In practical terms, GPs conceptualize the process of culturally sensitive care with an initiating exploration phase. At the beginning of and during physician-patient consultations, GPs should inquire about general patient preferences, including ideas, concerns and cultural expectations, in order to be able to take them into account throughout the further consultation and therapy recommendations. Continuously exploring whether certain aspects could bear cultural significance to individual patients, thereby demonstrating cultural awareness, may alleviate potential barriers during interactions with patients from diverse cultural backgrounds. The demonstrated openness of GPs contributes to both patient comfort and open communication between patient and provider.

*“For example, I do know that Muslim women, when they want to see a gynecologist, usually prefer a female practitioner, and so I try to show them that I know this. That it’s a cultural preference. [I say] ‘I would like to refer you to the gynecologist; we work with a number of gynecologists. Some are male, some are female. Do you have a preference?’ I never try to say, ‘I am going to refer you to a female,’ but I do ask. I show that I recognize this, while with someone else, just a Western woman, I say, ‘I am going to refer you to the gynecologist, to that doctor,’ without specifying that one is male and the other is female.”* (female GP, 39).

Following the phase of exploring patients’ cultural needs and preferences, GPs depict three distinct ways in how they deal with cultural expectations. A first possibility is that GPs take patients’ preferences into consideration and proceed to adapt their care provision (e.g. therapy recommendations, referrals, treatment) to patients’ needs. In this case, GPs are willing to change their “*normal*” behavior and tailor care to the patient.

Another possibility is that GPs are unwilling or unable to fully accommodate cultural preferences, potentially considering it undesirable, and instead opt to engage in deliberation and negotiation with the patient. Here the GP and patient will work together and negotiate in attempt to obtain a compromise between patients’ cultural preferences and the medical implications of these preferences. It is essential for both patient and provider to describe their expectations and to depict how they look at the matter at hand, within a safe environment.

*“that depends on the dialogue between [patient and provider], how that fairs. It’s certainly not ‘I’m absolutely never going to do that’, but it’s also not ‘I always respond to the patient’s request’. It really is trying, step by step, to write a story together.”* (male GP, 61).

Lastly, GPs could require patients to completely adapt or put aside their cultural preferences during the medical encounter. In this instance, GPs feel that they can only go so far, they are not willing or able to fully adapt to other cultures and, at a certain point, it is up to the patient.

This distinction in how GPs shape the process of culturally sensitive care varies by two main factors (see Fig. 1). On the one hand, the specific nature of the cultural preferences at hand influences GPs’ willingness or ability to tailor care according to these needs or preferences. As such, GPs might fully adapt to certain cultural expectations and depict a lot more resistance to other cultural preferences. On the other hand, the degree to which GPs adapt depends on their perceived responsibilities. Certain GPs attribute a larger role or responsibility to themselves to tailor care to patients’ cultural preferences than others.

### Specific cultural preferences

Firstly, GPs indicate a willingness to tailor care provision regarding specific cultural needs, whereas other cultural expectations cause GPs to feel more reluctant to adapt. Therefore, the process of culturally sensitive care is partially determined by inherent aspects of cultural preferences.

For instance, the vast majority of GPs in our sample readily prescribe medication in adherence to patients’ religious preferences regarding food and medication intake during Ramadan. A substantial number of patients believe that, during the fasting period, neither food, liquids nor medication can be consumed between sunrise and sunset. This presents specific challenges when GPs need to prescribe medications intended for e.g. thrice-daily consumption. While the intake of (necessary) medication is allowed during Ramadan, and certain alternatives are delineated in Islam, not all patients are aware of this. Consequently, GPs actively search for alternative methods, aligning with fasting regulations during Ramadan.

*“Our [previously discussed] patient, he must and he will take his pills, even if I have to call an Imam [religious leader]to convince him that he should take them. With other patients I have already adjusted medication schedules to Ramadan, where I say ‘yes take them right before and yes, right after breaking the fast and right before your fast starts again’. Or okay, we can use a different antibiotic with fewer doses, or if you’re not going to take your antibiotics properly, okay, then we won’t prescribe them. Um, then we’ll try it without antibiotics, because if you don’t take them properly then we’re just going to have more problems.”* (male GP, 29).

Interestingly, in the case of coping with dietary and medicinal restrictions during Ramadan, several participants indicated they feel more confident or capable to tailor care due to their knowledge of this concept. They emphasize that they, to a certain extent, know what Ramadan is, what it entails and how it may influence healthcare. One GP considered Ramadan to be accepted by the dominant culture. Therefore, GPs’ intercultural knowledge may substantially influence their confidence, capacity and willingness to deliver culturally sensitive care.

Another frequently mentioned theme is culturally specific illness perceptions. Interpretations of what it means to be sick, and subsequently, expressing symptoms and expectations regarding medication and treatments, can often substantially vary across different cultures. For example, GPs frequently reference encounters where ethnic minority patients more often expect medication, antibiotics, referrals or *“a scan of everything”*, whereas these are not required according to GPs’ assessment or guidelines. These cultural expectations can sometimes collide with GPs’ own cultural perceptions and framework and may result in GPs’ perception of minority patients as more “*theatrical”* and “*demanding”* in expressing their symptoms. Consequently, patients’ cultural expectations influence the process of culturally sensitive care, with GPs indicating a lesser inclination to readily accommodate these preferences compared to more widely recognized cultural needs, such as those observed during Ramadan. In cases involving culturally divergent interpretations of illness, GPs prioritize active listening, attempting to understand potential underlying issues, negotiating with patients, and taking appropriate action, rather than completely adapting to patients’ cultural preferences.

*“A different ethnicity also brings about different aspects, a different perception of illness, and different possibilities. I think sometimes maybe not enough thought goes into that. Physicians are also people coming from specific backgrounds. They are often white, Flemish doctors who originate from a particular setting, and I frequently sense that that setting provides little understanding or insight into what it is like to live in poverty, rely on benefits, or reside in a socially disadvantaged neighborhood. There seems to be little connection with these experiences, and the healthcare provided may not be adequately tailored or responsive to them, which is often overlooked. Physicians certainly take such factors into account, mostly on the most apparent aspects. I believe that there are many cultural differences that we are not aware of, that not everyone is familiar with, and that are not being explored or spontaneously discussed. In such cases, physicians cannot adapt their approach accordingly.”* (male GP, 28).

Similar to Ramadan, GPs also describe the importance of intercultural knowledge in addressing culturally specific perspectives on health and healthcare. In this context, however, more emphasis is placed on the lack of knowledge concerning certain interpretations, expressions and their origin. Without this knowledge, GPs may remain unaware of many cultural nuances and views on health, further hindering their capacity to tailor care provision accordingly. Consequently, intercultural knowledge may affect how cultural preferences shape GPs’ perceptions of the culturally sensitive care process. GPs with a heightened understanding of cultural values and expectations may find themselves better equipped and more adept in navigating cultural preferences, thereby mitigating potential negative impacts on the culturally sensitive care process.

Nevertheless, GPs also illustrate certain cultural values that result in considerably more frustrations or resistance towards adapting care provision. Ultimately, these cultural preferences may lead to a discernible cultural clash between the patient and the healthcare provider. The prevalent theme in this context revolves around cultural perspectives on gender, with GPs occasionally noting distinct gender inequalities in certain cultures. These cultural views can be exemplified by female patients expressing a preference for female care providers for intimate medical examinations, a preference GPs are very understanding of. However, gender-related cultural views can also manifest in both male and female patients expecting consultations with healthcare providers of the same gender, irrespective of any issues related to intimacy. Participants indicate how male patients might favor male physicians, viewing female physicians as inferior, while female patients may expect female physicians purely for their perceived comfort. Such preferences collide with GPs’ cultural beliefs regarding gender equality and may undermine their sense of professional competence. As GPs are not able “*to change their gender”*, the process of culturally sensitive care in these cases often necessitates a negotiation between patient and GP or requires patients to set aside cultural preferences and, therefore, fully adapt to GPs’ values. Alternatively, patients may opt to change their GP to better align with their preferences.

*“When it’s purely cultural, there are quite a few man-woman barriers that I experience. That is definitely something. Women who are actually denied care by their husbands or by themselves because it involves a male doctor, for instance. Women [colleagues] experience that a bit less, although some female physicians also tell me that they sometimes have less authority over male patients. The gender difference is certainly present.”* (male GP, 37).

Another gender-related issue highlighted by GPs in our sample does not pertain directly to the healthcare provider. GPs occasionally observe instances where female patients are frequently accompanied by their male spouses, who assume a dominant role during the consultation. In some cases, female patients are constrained by their husbands from undressing or adhering to the instructions provided by the GPs. Similar to preferences regarding GPs’ gender, these cultural inclinations collide with GPs’ beliefs regarding equality and emancipation. GPs report more difficulties with such cultural preferences, which may result in more reluctance to account for these cultural values in providing care. In such instances, GPs may attempt to persuade female patients to attend consultations alone, thereby shifting the focus of culturally sensitive care towards modifying patient behavior.

Lastly, as highlighted by several GPs, a general boundary in the process of providing culturally sensitive care occurs when specific cultural requests are not scientifically based or, more importantly, not medically rational or “*scientifically justifiable”*. For instance, patients may ask for specific scans or investigations that are medically unnecessary or not recommended, or demand antibiotics or potent painkillers without medical justification. Therefore, variations in how specific cultural preferences influence the process of culturally sensitive care provisions may be explained by the extent to which patients’ preferences challenge the professional competence and values of GPs, as well as by the potential risks they pose to patients’ well-being.

### Perceived responsibilities

In addition to the nature of cultural preferences, another pivotal factor shaping the process of culturally sensitive care is the responsibilities GPs ascribe to themselves. The perceived responsibilities of GPs’ constitute a recurring theme in our data, and while these responsibilities often exhibit variation among participants, general patterns have also emerged.

Throughout our interviews, GPs consistently indicate a sense of responsibility in exploring patients’ cultural needs and expectations (i.e. engaging with the initiating exploration phase). Subsequently, they feel responsible for adapting care provision accordingly. Moreover, according to the respondents, it is the GPs’ responsibility to establish a safe environment for patients to express their preferences and to provide comprehensive advice regarding medication, treatments and the consequences of not complying with GPs’ recommendations.

Contrastingly, the prevailing viewpoint among most GPs asserts that adherence to medication or therapy recommendations is entirely and exclusively the patient’s responsibility. GPs consider the neglect of their recommendations due to cultural beliefs as beyond their control and, therefore, they do not regard it as their responsibility.

*“I may be a bit naive, but we are an advisory council. Ultimately, a patient comes for advice, not to undergo what we decide. I find that incorrect. If the patients’ goal is to get better, I will explain how. ‘If you come with a complaint and you want to heal from it, these are the options, and if you refuse those options, that is your choice.’”* (male GP, 37).

Notably, these insights appear to contradict the previously expressed openness of GPs’ to adapting to preferences related to medication during Ramadan. While GPs demonstrate a readiness to engage in discussions about preferences, deviating from established treatments based on cultural values may result in a diminished sense of responsibility among GPs. This underscores the need to consistently explore and address patients’ cultural needs and preferences throughout the course of treatment and to engage in shared decision-making processes.

Despite general agreement regarding perceived responsibilities, several instances arose depicting individual differences. Therefore, even within specific cultural preferences where most GPs feel responsible in a uniform or similar way, individual GPs may display varying senses of responsibility. These variations, in turn, influence the shaping of the process of culturally sensitive care in distinct ways.

GPs feeling more responsible in providing culturally sensitive care, might adapt their behavior more frequently and may less often require negotiation or patients to completely adapt their cultural expectations and preferences. For instance, one participant emphasizes the overall responsibility of GPs to adapt their approach, regardless of specific cultural needs.

*“You must adapt to your patient, of course. If you have a patient who is intellectually disabled or who is less proficient in Dutch than someone else, you must adjust your language. If you have someone with very poor adherence to therapy, you may propose a different therapy where adherence is less essential than for someone you know will stick to it well.”* (female GP, 29).

Conversely, another example illustrates the reverse effect of GPs perceived responsibility. Whereas the majority of GPs in our sample expressed a willingness to accommodate patients’ cultural preferences, such as adjusting medication intake during Ramadan, one GP does not consider herself responsible for such treatment customization. As a result, this absence of a sense of responsibility steers the trajectory of culturally sensitive care towards the expectation that patients should adapt.

*“It’s not my problem, it’s the person in front of you who has a problem. He doesn’t pass his problem on to me. I’m not going to go through the infection myself. If I have an infection and antibiotics are necessary, I’d take my antibiotics. If it’s for cultural reasons that it can’t be done, I don’t know. I’ve never been in a situation like that before. But I would rather have the idea: not my problem. I give you the advice and you do with it what you want. And if you don’t see it as a solution, then don’t come and say, ‘I can’t take antibiotics three times a day [during Ramadan], give me another solution.’ If I say there’s no other solution, then there is no other solution.”* (female GP, 51).

One possible explanation for diverging perceptions of responsibility within our sample is distinct outlooks on diversity and multiculturalism, clearly indicated by varying levels of cultural openness among GPs in our study. Specifically, we observe variations regarding their characterization of interactions with patients from different ethnic backgrounds. Some GPs define these interactions as more interesting and engaging, asserting that valuable information can be learned from understanding the thought processes of individuals from other cultural backgrounds. Other participants illustrate how these encounters can be an “*enrichment”* by learning how others live and experience things. In this perspective, intercultural care encounters are viewed as an opportunity rather than an obstacle, reflecting cultural openness. However, other participants depict multiculturalism in general practice as more challenging, frustrating and burdensome.

*“Do we need to completely adapt ourselves to the person in front of us, or do we work according to what we are used to? If you experience a language barrier with a person, that person would definitely not, if there’s a language barrier, receive the same quality treatment you would give to someone who speaks the same language as you. But can you avoid that? That is the question. Should you? Preferably yes, but you will have to invest very much.”* (female GP, 51).

In these cases, emphasis is placed on a required investment to cope with different cultures and values, rather than an opportunity to learn more about other cultures. One GP describes these encounters as “*confusing”* and “*frightening”*, referring to a fear of losing one’s own cultural identity while adapting to other cultures.

These different outlooks on diversity and senses of responsibility can also be interpreted as varying levels of cultural empathy. For instance, our data illustrates how certain GPs appreciate the value of understanding patients’ perspectives, whereas others do not. Such empathic attitudes are likely to shape GPs’ perceptions of culturally sensitive care and sense of responsibility in adapting care tailored to patients’ cultural values and preferences.

## Discussion

### Implications of findings

This study aimed to explore GPs’ perceptions regarding culturally sensitive care, including their intentions, willingness and perceived responsibilities. Utilizing in-depth interviews with Flemish GPs, our findings reveal that culturally sensitive care is conceptualized as a multifaceted process of pursuit, in line with Campinha-Bacote’s [19] care model defining it as “*the ongoing process in which the healthcare provider continuously strives to achieve the ability to effectively work within the context of the client*” ([19], p. 181). Additionally, consistent with previous studies [57], our findings underscore the importance of the GP’s context in achieving culturally sensitive care, with GPs indicating they strive to achieve cultural sensitivity despite the inherent contextual restrictions present in general practice settings.

Furthermore, GPs describe this process as initiating with an exploration phase, requiring them to explore patients’ cultural needs, preferences and expectations. Addressing such preferences is essential at the start of consultations, and throughout the entire interaction and treatment recommendations. This approach allows GPs to differentiate treatment, marked by equal amounts of respect and interest for each patient, striving to achieve equitable care outcomes. The process of culturally sensitive care is further shaped by two pivotal factors: patients’ specific cultural expectations and GPs’ perceived responsibilities.

Patients’ cultural preferences most often mentioned by GPs in our sample, such as dietary restrictions during Ramadan and gender preferences, underscore participants’ tendency to focus on Muslim patients and those of Moroccan-Maghreb or Turkish backgrounds when discussing the notion of culturally sensitive care. Whilst these groups do constitute two of the largest ethnic minority groups in Belgium, other groups or ethnicities were mentioned notably less often. Similar to findings by Claeys et al. [21] and Wets et al. [62], our data suggests a risk of narrowing cultural sensitivity to religious distinctions, a reductionist focus on select ethnic groups and “othering”. This marginalization of ethnic minority patient groups, viewing them as ‘the other’, might reinforce social inequalities, albeit often unintentionally, and neglects the intricate nature of culture in healthcare [63].

GPs further demonstrate how their competence and confidence in managing patients’ cultural preferences are augmented by increased intercultural knowledge. Conversely, a lack of familiarity with specific cultural preferences results in diminished capability or confidence in accommodating these preferences. Additionally, when GPs displayed less knowledge or understanding of cultural differences, they more often tended to resort to using stereotypes. The use of stereotypes negatively influences intercultural communication processes and contributes to healthcare disparities by fostering unequal treatment [15, 64]. However, as for instance Schouten et al. [41] emphasized, heightened intercultural knowledge might inadvertently also lead to stereotyping, as it may overlook within-group distinctions, necessitating a deeper understanding of the mechanics of stereotyping. Our findings further demonstrate the risk of wrongfully assuming one can know another culture, viewing it as something static, concrete and applicable to all members of an ethnic group [41, 63]. Therefore, in line with previous recommendations [19, 23, 44, 55], integrating lifelong learning as a central component of intervention and training programs is crucial to address these obstacles and promote continual improvement in patient-centeredness and cultural sensitivity among healthcare providers.

GPs’ increased reluctance to adapt care provision when they feel less confident or knowledgeable about cultural differences opposes the imperative of curiosity and desire to explore patients’ preferences and their origins, as advocated in cultural sensitivity models [19, 32, 41, 65]. This becomes particularly evident in discussions surrounding cultural views on gender. The perceived lack of emancipation in certain cultures and preferences for gender concordant consultations may lead to a collision with GPs’ cultural values and a perceived lack of acknowledgement of physicians’ professional competence. Such discrepancies, previously studied as cultural distance between patient and provider [66], often lead to a lack of understanding and, consequently, resistance in GPs to explore and discuss patients’ cultural preferences, thereby hindering cultural sensitivity. Cultural distance has already been shown to negatively influence the quality of care [66, 67], which further underscores the necessity of exploration, cultural reflexivity and critical consciousness during intercultural care encounters.

The second pivotal factor guiding the culturally sensitive care process, pertains to GPs’ perceived responsibilities. Our study identified a general sense of responsibility among the sampled GPs, particularly concerning the exploration of patients’ needs and expectations, as well as the establishment of a safe healthcare environment. This observation aligns with the assessment by Dauvrin and Lorant [5] regarding healthcare providers’ responsibility for comprehensive information provision. However, a predominant viewpoint among our sample was that adherence to medication and therapy recommendations falls entirely on the patients, despite the importance of shared decision-making and evidence indicating the crucial role of providers in therapy adherence [32, 68–70]. Many GPs perceived their role merely as an “*advisory board*” in therapy recommendations, placing the responsibility on patients to adhere or not adhere to given recommendations. Nevertheless, evidence suggests that healthcare providers’ cultural sensitivity is associated with patients’ adherence, highlighting the importance of shared decision-making and effective, patient-centered communication strategies in recommending therapy.

Participants also displayed varying senses of responsibility, as evidenced by their willingness to adapt to Ramadan requirements. This variability in sense of responsibility may be attributed to the different attitudes of GPs towards diversity, similar to findings by Duveau et al. [58]. While some perceive diversity as an opportunity for enrichment, others view it with apprehension, feeling as though their own moral values, rooted in the medical profession, are jeopardized by what they may perceive as inferior cultural values. Appreciation of and openness towards other cultures are fundamental prerequisites for cultural sensitivity [20, 23]. Therefore, it is highly plausible that cultural openness influences GPs’ perceived responsibility and, consequently, the customization of care to meet patients’ cultural needs.

Moreover, variations in the sense of responsibility may also be explained by GPs’ level of empathy, another concept widely acknowledged as fundamental to cultural sensitivity [32, 71, 72]. Empathy has been associated with increased effectiveness in intercultural care [51], more patient trust in providers [32, 71] and a heightened sense of responsibility [73, 74]. Consequently, our data underscores the significant importance of empathy in both GPs’ perceived responsibilities and the overall provision of culturally sensitive care. Additionally, cultural openness and empathy can reciprocally influence each other [75], reinforcing the importance of fostering both cultural openness and empathy within healthcare settings.

Ultimately, both patients’ cultural preferences and GPs’ perceived responsibilities guide the process towards three distinct possible outcomes. These outcomes range from complete adaptation to patients’ cultural requirements driven by a high sense of responsibility, through negotiation and compromise, to a paternalistic approach where patients are expected to conform to GPs’ values and expectations.

Based on this distinction, guided by patients’ specific cultural needs and GPs’ perceived responsibilities, we propose a typology that consists of three types of GPs in the provision of culturally sensitive care. It is important to note that these categories should not be perceived as rigid or deterministic; rather, they are dynamic and interconnected, evolving through ongoing interactions. This characterization aligns with previous studies employing social positioning theory to delineate health professionals’ roles [76].

The first type encompasses GPs distinguished by genuine cultural sensitivity. They possess comprehensive knowledge of cultural disparities, and a genuine curiosity to explore patients’ cultural needs and values. Moreover, they exhibit empathy and an openness to diverse cultures. Recognizing the pitfalls of stereotyping, they feel a heightened sense of responsibility to accommodate patients’ expectations and tailor care provision to meet their individual needs. Notably, our observations suggest that younger GPs, as well as those more frequently encountering ethnically diverse patients, tend to align more closely with this type, demonstrating higher levels of cultural sensitivity and a greater openness to cultural diversity.

The second type encompasses surface-level culturally sensitive providers who, while customizing care provision to some extent, lack sustained curiosity about cultural nuances. Although they possess some knowledge of cultural differences and their impact on the care process, it tends to be rather superficial, and they often fail to grasp culture as a dynamic and multi-dimensional concept that encompasses more than just the beliefs and practices of specific groups.

Lastly, the third type represents GPs who perceive diversity as a threat. These practitioners exhibit minimal cultural empathy and are resistant to deviate from biomedical models to tailor care provision to cultural needs, consequently burdening patients with the responsibility of adaptation. They frequently individualize patient choices, such as abstaining from medication during Ramadan, without acknowledging the broader cultural context impacting health and illness perceptions.

### Limitations

While our study provides valuable insights into GPs’ perceptions of culturally sensitive care, several limitations should be acknowledged. Firstly, our study relied on self-reported perceptions and experiences of GPs, which may be subject to social desirability bias or recall bias. Incorporating observations of actual patient-provider interactions or employing mixed-methods approaches, including mystery patients, which have proven to be commendable approaches in various fields [77, 78], could provide a more comprehensive understanding of culturally sensitive care practices.

Furthermore, our study predominantly explored cultural sensitivity in the context of ethnicity, overlooking other dimensions of diversity such as socioeconomic status, sexual orientation, or disability, as this would exceed the scope of the current study. Future studies should adopt a diversity sensitive approach to examine how multiple aspects of patients’ identities intersect and influence healthcare interactions. Additionally, participants often referenced Islamic and religious traditions, potentially narrowing the conceptualization of culture. Subsequent studies should examine whether similar patterns emerge among GPs interacting with diverse ethnicities beyond those of Islamic faith to enhance our comprehension of cultural sensitivity in healthcare.

Moreover, our study primarily focused on GPs’ perspectives, overlooking the views of other healthcare stakeholders such as patients, nurses, or specialists. Future research should adopt a multi-stakeholder approach to gain a more holistic understanding of cultural sensitivity in healthcare settings.

Lastly, our participant sample, the interviewer and entire research team exclusively consisted of people of Flemish, ethnic majority descent. While this composition facilitated linguistic fluency and cultural familiarity, it also poses risks of biased perspectives and limited understanding of broader ethnic and cultural contexts in healthcare. Diverse teams offer innovative perspectives and help identify and address knowledge gaps, particularly when researching culture and equity [79]. Moving forward, prioritizing diversity within research teams is important for fostering inclusivity and validity in efforts to understand and mitigate healthcare disparities.

### Future research directions

In terms of future research directions, longitudinal studies could explore the effectiveness of cultural sensitivity training programs or interventions aimed at enhancing healthcare providers’ cultural awareness, reflexivity, and empathy, and their impact on healthcare outcomes over time. Moreover, comparative studies conducted across various healthcare systems or cultural contexts could offer valuable insights into the applicability and transferability of culturally sensitive care practices, including the relevance of our coding scheme in diverse ethnic settings. Investigating the presence or absence of our suggested typologies in different contexts would provide further understanding of their generalizability and utility. Furthermore, delving into the underlying factors driving individuals to adopt specific typologies, such as those identified in our study (e.g., physicians who strongly believe in the validity and superiority of biomedical models, derive status from their professional identity, and perceive cultural diversity as a threat are more inclined to fall into the latter typology), would offer deeper insights into the dynamics of culturally sensitive care provision.

Overall, addressing these limitations and pursuing future research directions will contribute to the advancement of knowledge in the field of culturally sensitive care and ultimately enhance the quality of healthcare delivery for diverse patient populations.

## Conclusion

In conclusion, our study sheds light on GPs’ perceptions of culturally sensitive care, revealing it as a multifaceted process of pursuit shaped by patients’ specific cultural preferences and GPs’ perceived responsibilities. Despite commendable intentions of some GPs to accommodate cultural diversity, challenges persist, including the risk of stereotyping, cultural distance, and varying levels of empathy and sense of responsibility among healthcare providers, hindering the process of culturally sensitive care provision.

By delineating three typologies of GPs based on their approach to culturally sensitive care, our study highlights the importance of genuine cultural sensitivity, sustained curiosity about cultural influences, and empathy in healthcare practice. Recognizing and addressing these typologies may be crucial for fostering equitable and patient-centered care.

Moving forward, it is imperative to address the limitations of our study and pursue future research directions to further understand and enhance culturally sensitive care practices. By doing so, we can strive towards healthcare practices that are genuinely inclusive, responsive, and respectful of diverse patient needs and preferences.

## Data Availability

The datasets used and/or analyzed during the current study are available from the corresponding author on reasonable request.

## Abbreviations

GP: General Practitioner
